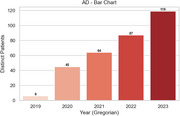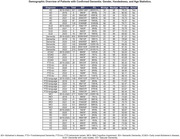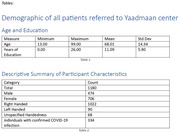# Yaadmaan Dataset in Iran: Bridging a gap in Dementia Research

**DOI:** 10.1002/alz70860_098768

**Published:** 2025-12-23

**Authors:** Mohammad Zamani, Maryam Noroozian, Yaadmaan Institute for Brain, Cognition and Memory Studies Research Group

**Affiliations:** ^1^ Cognitive Neurology, Dementia and Neuropsychiatry Research Center, Tehran University of Medical Sciences, Tehran, Tehran, Iran (Islamic Republic of); ^2^ Yaadmaan Institute for Brain, Cognition and Memory Studies, Tehran, Tehran, Iran (Islamic Republic of); ^3^ Tehran University of Medical Sciences (TUMS), Tehran, Tehran, Iran (Islamic Republic of); ^4^ Cognitive Neurology, Dementia and Neuropsychiatry Research Center (CNNRC), Tehran, Tehran, Iran (Islamic Republic of)

## Abstract

**Background:**

As populations age worldwide, including in Iran, and encounter significant socioeconomic challenges, dementia rates may be climbing. Some projections suggest the Middle East and North Africa could face a marked increase—potentially approaching 357%—though exact figures remain uncertain. Despite the growing disease burden, this region lacks a critical lack of cohesive, high‐quality data. This deficit hampers effective public health strategies, early diagnosis, targeted interventions, and robust research. Although location and socioeconomic barriers may limit attendance for some individuals, the Yaadmaan Institution—serving as a major referral center for patients from across Iran—provides a near‐ideal setting for establishing a clinical dementia database through this ongoing retrospective project.

**Method:**

A multifaceted approach is employed to create a structured, secure database. An application programming interface (API) linked to a MongoDB server collects and organizes patient information—demographic data, family history, risk factors, medication profiles, and neuropsychological assessments (MoCA, MMSE, FAB, etc.). A multidisciplinary team—including neurologists, psychiatrists, cognitive scientists, geriatricians, and data management specialists—was trained to accurately extract and input data from paper records. Data entry is ongoing, focusing on at least the past five years of patient visits at Yaadmaan. Strict ethical standards, including de‐ identification procedures and controlled user access (via a whitelist of approved IPs), ensure patient confidentiality.

**Result:**

A total of 854 individuals with a dementia diagnosis have been identified in our database. Alzheimer's disease (AD) is the most common subtype (321), followed by FTD‐language (148), FTD‐ bv (28), SD (4), DLB (27), EOAD (17), MCI (277), and VD (32). Although FTD‐bv is believed to be more prevalent, its psychiatric‐like symptoms may lead to misdiagnosis or delayed neurology referrals, resulting in higher FTD‐language documentation. Additionally, the lower referral numbers from 2019 to 2021—likely attributable to COVID‐19 lockdowns—should be interpreted with caution.

**Conclusion:**

This database stands to enhance evidence‐based policymaking, facilitate early detection and intervention, and improve patient care quality. By leveraging the unique referral‐based population at Yaadmaan, the platform will foster cross‐disciplinary collaborations and inform national and regional strategies for dementia prevention, diagnosis, and management in Iran and the broader Middle East.